# Measurement invariance of the PROMIS emotional distress and subjective well-being domains among autistic and General Population adolescents

**DOI:** 10.1007/s11136-024-03742-9

**Published:** 2024-07-30

**Authors:** Elizabeth A. Kaplan-Kahn, Rachel M. Benecke, Whitney Guthrie, Benjamin E. Yerys, Laura Graham Holmes, Judith S. Miller

**Affiliations:** 1 Center for Autism Research, Children’s Hospital of Philadelphia, Philadelphia, PA USA; 2 College of Education and Human Development, Temple University, Philadelphia, PA USA; 3 Perelman School of Medicine, University of Pennsylvania, Philadelphia, PA USA; 4 Silberman School of Social Work at Hunter College, City University of New York, New York, NY USA

**Keywords:** Autism, Adolescence, Measurement invariance, PROMIS, Self-report, Patient-reported outcomes

## Abstract

**Purpose:**

Quality of life (QoL) is identified as a clinical and research priority by the autistic community. Researchers have the responsibility to ensure that instruments used to measure QoL do so reliably and accurately among autistic participants.

**Methods:**

Our study evaluated measurement invariance of Emotional Distress (Depression, Anxiety, Anger, Psychological Stress) and Subjective Well-Being (Life Satisfaction, Positive Affect, and Meaning & Purpose) scales of the Patient-Reported Outcomes Measurement Information System (PROMIS) among groups of autistic (N=140, n per scale=132–140) and general population (N=1,224, n per scale=406–411) teenagers (14–17 years). These scales were included in the PROMIS Autism Battery-Lifespan, which uses PROMIS scales to measure QoL domains most relevant for autistic people.

**Results:**

Multi-group confirmatory factor analyses using permutation tests demonstrated that Depression and Positive Affect scales exhibited scalar invariance between groups, indicating that scores can be meaningfully compared across autistic and general population teens. Anger and Psychological Stress scales demonstrated metric invariance between groups, indicating that these scales measure the same latent trait in both groups, but group comparisons are not supported.

**Conclusion:**

We provide guidance as to how these scales can be used in psychometrically supported ways to capture constructs relevant for understanding QoL among autistic teens.

**Supplementary Information:**

The online version contains supplementary material available at 10.1007/s11136-024-03742-9.

## Abstract (fr)

Quality of life is an important outcome for autistic people and their families. However, many quality of life scales have not been tested to make sure they accurately measure quality of life among autistic people. It is important to make sure that quality of life measures works similarly among the autistic population. The goal of our study is to test whether scales on a common quality of life measure – the Patient-Reported Outcomes Measurement Information System (PROMIS) – measures quality of life in the same way between autistic and general population adolescents. We found that some of the PROMIS scales worked the same way for autistic and general population adolescents and others did not. These results help researchers confidently use the PROMIS scales to measure different aspects of quality of life among autistic youth.

## Introduction

Autistic^1^ advocates and their families prioritize research and clinical services focused on outcomes [3–6]. Prominent among these outcomes is quality of life (QoL), which the World Health Organization defines as “an individual’s perception of their position in life in the context of the culture and value systems in which they live and in relation to their goals, expectations, standards, and concerns” [7]. Maximizing QoL among autistic individuals is often identified as a goal across a wide range of disciplines in autism research [8]. Agencies and organizations at national (e.g., the Interagency Autism Coordinating Committee) and international (e.g., the World Health Organization) levels have also emphasized large gaps in our current understanding of how support services and care models can increase QoL among autistic people [9]. However, to maximize QoL among autistic people, researchers need to ensure that they are using psychometrically reliable and valid tools to measure autistic QoL.

Psychometric validation of QoL measures is critical when considering their use in autistic populations. If a measure’s functioning has not been tested within autistic populations, autism researchers cannot assume they are accurately measuring QoL. Making the assumption that a measure functions the same way across populations without evidence poses a threat to the measure’s validity, impeding its clinical utility [10]. In qualitative studies, autistic individuals and their parents discussed the items of popular QoL measures and indicated multiple themes impacting the validity of their use within autistic populations, including emotional vocabulary and misinterpretation of items [11]. Thus, there is a clear need to test the psychometric properties of QoL instruments developed for use in the general population in order to confidently use these measures in autistic populations.

Literature examining psychometric properties of QoL measures among autistic individuals is largely focused on adults [12]. Findings from this nascent literature demonstrate potentially questionable psychometric support for popular QoL measures developed for the general population. For example, the World Health Organization Quality of Life-Brief Version (WHOQOL-BREF) [13] demonstrated mixed fit indices in a sample of autistic adults and required four iterations of post-hoc modifications to achieve acceptable fit [14]. Psychometric support for the autism-specific supplement to the WHOQOL-BREF, the Autism Spectrum Quality of Life [14, 15], has also been mixed [16]. The Patient-Reported Outcomes Measurement Information System Global Health survey (PROMIS Global-10), demonstrated promising psychometric properties, and minimal differential item functioning across subgroups of autistic adults [17]. These results provide the evidence necessary to confidently use the PROMIS Global-10 as a reliable and valid indicator of QoL in autistic adults. However, this work did not explore whether such a measure performed similarly across autistic and general population groups. Therefore, for research seeking to compare QoL scores across groups of participants, open questions remain about whether the PROMIS scales demonstrate measurement invariance across autistic and general population groups.

When comparing scores from any given scale across two or more groups, researchers make an implicit, yet consequential, assumption that the scale measures the same construct in the same way across the groups. This assumption is known as measurement invariance, which is defined as “whether or not, under different conditions of observing and studying phenomena, measurement operations yield measures of the same attribute” [18]. Comparisons and interpretations of scores between groups are only meaningful if the scale demonstrates measurement invariance [19, 20]. Research has explored measurement invariance across autistic and general population groups for domains such as cognitive functioning [21–23], behavioral concerns [24], sleep impairment [25], and depression [26, 27]. Given the burgeoning emphasis on incorporating QoL outcomes into autism research, it is crucial to evaluate measurement invariance in QoL measures.

To measure autistic QoL, Graham Holmes et al. (2020) leveraged patient-reported outcome measures from the National Institutes of Health (NIH) [28] to curate a specialized battery for assessing QoL across the lifespan for individuals on the autism spectrum: the PROMIS Autism Battery – Lifespan (PAB-L). Each measure includes scales assessing various domains of QoL, including health, emotional distress, subjective well-being, and social functioning. The PAB-L demonstrated good reliability, feasibility, and acceptability among a large (*N* = 912) sample of autistic individuals and their families [29]. Despite these strengths, it remains unclear whether items on the PAB-L contribute to the measurement of QoL in the same way across autistic and general populations.

The primary objective of the current study was to evaluate the measurement invariance of the PAB-L Emotional Distress (Depression, Anger, Anxiety, Psychological Stress) and Subjective Well-Being (Life Satisfaction, Positive Affect, and Meaning & Purpose) scales among autistic and general population teens. Using existing data from the original PAB-L study [29] and from publicly available PROMIS pediatric scores from a nationally representative sample of participants [30], we conducted secondary data analyses using multi-group confirmatory factor analyses (CFA) to assess for measurement invariance across autistic and general population adolescents. We focused our analyses on self-report because we were primarily interested in the measurement of *lived experience* of QoL, which is best captured by self-report.

## Methods

### Participants

Teenagers and their parents participated via online surveys. Parents reported on their teen’s demographic information in both autistic and general population groups. Participants were considered eligible if they were between the ages of 14 and 17 years old. Demographic characteristics, including age, gender, race, and ethnicity, are reported for participants with available data for at least one PROMIS scale (Table 1). All recruitment and study procedures were approved by the institutional review board at the Children’s Hospital of Philadelphia.

**Table 1:** Demographic information

Variable	Autistic Group	General Population Group
Totals	140 (132–140 per scale)	1,224 (406–411 per scale)
Age		
14	35 (25.71%)	293 (23.94%)
15	36 (25.71%)	313 (25.57%)
16	36 (25.71%)	321 (26.23%)
17	33 (23.57%)	297 (24.26%)
Gender		
Female	37 (26.43%)	604 (49.35%)
Male	100 (71.43%)	620 (50.65%)
Transgender	3 (2.14%)	N/A
Race		
Asian	0 (0%)	46 (3.76%)
Black	5 (3.57%)	114 (9.31%)
Native American	0 (0%)	5 (0.41%)
White	130 (92.86%)	924 (75.49%)
Other or more than one race	5 (3.57%)	165 (13.48%)
Missing	0 (0%)	1 (0.08%)
Ethnicity		
Hispanic or Latino/a	9 (6.43%)	165 (13.48%)
Not Hispanic or Latino/a	131 (93.57%)	1,058 (86.44%)
Missing	0 (0%)	1 (0.08%)

### Autistic sample

For our autistic sample, we used adolescent self-report data from Graham Holmes et al. (2020). Participants were recruited via the Children’s Hospital of Philadelphia or the Interactive Autism Network between November 2017 and June 2018. 140 participants responded to at least one PROMIS scale (Table 2). The sample of autistic adolescents was well distributed across ages, predominantly white (92.86%) and non-Hispanic/Latino (93.57%). Approximately three times as many autistic participants identified as male (*n* = 100; 71.43%) than identified as female (*n* = 37; 26.43%), and three autistic adolescents identified as transgender (Table 1).

**Table 2:** Domain summaries by group

Scale	Autistic Group				General Population Group		
	*n*	Cronbach’s alpha	*M (SD)*		*n*	Cronbach’s alpha	*M (SD)*
Emotional Distress							
Anger	132	0.89	51.13 (11.10)		406	0.91	46.15 (10.52)
Anxiety	139	0.93	53.08 (10.90)		406	0.92	44.29 (9.77)
Depressive symptoms	139	0.95	53.42 (11.43)		411	0.95	46.85 (10.40)
Psychological stress	138	0.95	59.06 (9.70)		407	0.94	52.11 (9.74)
Subjective Well-Being							
Life satisfaction	139	0.96	43.63 (10.17)		407	0.96	46.96 (9.43)
Meaning and purpose	138	0.94	41.67 (10.10)		411	0.93	48.60 (9.12)
Positive affect	140	0.93	45.64 (8.95)		406	0.94	48.12 (8.89)

*Note* M = Mean, SD = Standard Deviation

#### General population sample

Our comparison sample comes from a nationally representative normative sample of the PROMIS [30]. Participants were recruited by GfK Knowledge Panel, an extant online probability panel of participants in the United States. For adolescent self-report, 1,627 participants responded to at least one PROMIS scale, with approximately 400 participants responding per scale (Table 2). Like the autistic sample, general population teens were distributed evenly across the range of ages. Recruitment for the general population sample aimed to be representative of the United States population, reflected in a balanced gender ratio (50.52% identifying as male) and increased racial and ethnic diversity, albeit still mostly white (76.58%) and non-Hispanic/Latino (86.11%). No response option for identifying as transgender was included for the general population sample (Table 1).

### Measure

The PROMIS pediatric scales are measures of QoL, publicly available through the NIH [31]. Each scale is unidimensional [32–38], assessing a single factor driving QoL, and can be administered individually or as part of a wider QoL battery. PROMIS scales were validated on representative samples of the U.S. population [39]. Responses for each scale item range from 1 to 5, with higher scores corresponding to higher endorsement of the construct being measured. Most PROMIS scales are available in three formats: parent proxy (for 5–17-year-olds), pediatric self-report (for 8–17-year-olds), and adult self-report (for 18 + year-olds). Many scales include short forms, which consist of 4 to 8 items and are scored through the Assessment Center (www.assessmentcenter.net) [40]. Short forms of all the self-report scales used in the current study have demonstrated no meaningful differential item functioning as a function of gender [32–37]; thus, while autistic and general population teens differed in their gender ratios (Table 1), prior evidence supports the conclusion that the scales perform similarly across genders.

The present study evaluated adolescent self-report PROMIS scales related to the domains of Emotional Distress (Anger, Anxiety, Depression, Psychological Stress scales) and Subjective Well-Being (Life Satisfaction, Positive Affect, Meaning & Purpose scales). Though the Emotional Distress and Subjective Well-Being domains do not represent higher order factors, they are drawn from past literature and theoretical judgement of the PROMIS scales [29, 39, 41]. We evaluated the short form version of each scale, consisting of 5 items for Anger and 8 items for all other scales (i.e., Anxiety, Depression, Psychological Stress, Life Satisfaction, Positive Affect, and Meaning & Purpose).

### Data analysis

We first fit CFA models to the baseline PROMIS models for the combined group of participants using the *lavaan* package in R [42]. Models were identified by setting factor variance to 1. Given the ordinal nature of the PROMIS data (reported on a Likert scale), we used the robust diagonally weighted least squares estimator and pairwise deletion was used to handle missing values. Due to limitations to using standard fit indices when using diagonally weighted least squares estimation [43], we evaluated model fit using robust categorical maximum-likelihood estimators including Comparative Fit Index (CFI_cML_), Tucker-Lewis Index (TLI_cML_), and Standardized Root Mean Squared Residual (SRMR_cML_). These fit indices were evaluated in assessing adequate model fit, including: CFI_cML_ and TLI_cML_ values of ≥ 0.95, and SRMR_cML_ values of ≤ 0.08 [44, 45]. Root Mean Square Error of Approximation (RMSEA) values exhibit large standard errors in models with few items and factors [46], and above-threshold RMSEA values are common among the PROMIS pediatric scales, even among their validation samples [33, 35, 47]; thus, given that each PROMIS scale is unidimensional and short forms of each scale were used in the current study, we did not evaluate model fit using RMSEA.

Next, to evaluate whether measurement of QoL domains varied between the general population sample and the autistic sample, we fit a series of increasingly restrictive multigroup CFA models to the data [45, 48] using *lavaan* [42] and *semTools* [49, 50]. Multigroup CFA models were conducted for each scale separately. First, multigroup CFA models were tested for *configural* invariance by allowing all factor loadings and item intercepts to vary freely for both groups. This analysis examined whether the overall factor structure of the measure fit well for both groups. Second, *metric* invariance was evaluated by testing for differences in fit between configural CFA models and models in which factor loadings were constrained to be equivalent across groups while allowing item intercepts to vary freely. This analysis tested whether the pattern of item loadings was equivalent across groups. Third, we tested the CFA models for *scalar* invariance by testing for differences in fit between the metric CFA models and models in which both factor loadings and item intercepts were constrained to be equivalent across groups.

Recommendations for criteria to evaluate changes in model fit within invariance models vary widely and depend on several relevant factors such as sample size, difference in sample size between the groups, and level of invariance [44, 46, 51, 52]. Given these limitations, we used permutation tests [49] to evaluate the hypothesis of measurement invariance and determine whether the ∆χ^2^ and the ∆CFI between the increasingly restrictive models were significant. As reported in Jorgensen et al., 2018, permutation tests provide better Type-I error control than commonly used benchmarks. We generated null distributions for all measurement invariance tests based on 1,000 permutated datasets, and non-significant *p*-values (≥ 0.01) indicated evidence of measurement invariance (i.e., no significant decrease in model fit between increasingly restrictive models). To establish invariance, we required both ∆χ^2^ and ∆CFI to be non-significant.

There were cases on the Anxiety and Positive Affect Scales where a very small number of participants endorsed a particular response for an item, such that after pairwise deletion and permutation, no instances of that response was present in one group or the other. This occurred in both the autistic and general population teen samples. To address this issue, we collapsed adjacent response categories (Table S1) for these scales when testing for measurement invariance [53, 54].

When configural invariance was not established based on at least one significant permuted fit indicator, model modification indices were subsequently examined for each scale and modifications to the model were implemented after ensuring a theoretical justification for the modification. In line with past literature testing measurement invariance between autistic and nonautistic groups [22], the modified models were then used in subsequent measurement invariance steps (when modifications were required, table S2).

## Results

### Internal reliability

Across both autistic and general population teens, the PROMIS scales demonstrated good to excellent internal consistency (alpha range 0.89–0.96, Table 2). The Anger scale demonstrated the lowest Cronbach’s alpha values for both groups, which is expected given that this scale had the fewest items.

### Confirmatory factor analysis

Initial baseline model fits for the PROMIS scales for the combined sample were adequate to excellent for all scales across all fit indices (Table 3). Likewise, the fits of the scale models in the autistic and general population samples were adequate to excellent across all fit indices (Table 4).

**Table 3:** Confirmatory factor analysis model results – combined sample

Model	*X* ^2^	df	*p*	CFI_cML_	TLI_cML_	SRMR_cML_
*Emotional Distress*						
Anger	102.737	5	< 0.001	0.948	0.895	0.039
Anxiety	124.666	20	< 0.001	0.940	0.916	0.032
Depressive Symptoms	125.392	19	< 0.001	0.964	0.946	0.020
Psychological Stress	104.996	19	< 0.001	0.978	0.968	0.021
*Subjective Well-Being*						
Life Satisfaction	242.282	19	< 0.001	0.954	0.933	0.026
Meaning & Purpose	216.716	19	< 0.001	0.942	0.915	0.035
Positive Affect	180.392	18	< 0.001	0.948	0.919	0.032

*Note* df = Degrees of Freedom, CFI = Comparative Fit Index, TLI = Tucker-Lewis Index, SRMR = Standardized Root Mean Squared Residual, cML = Categorical Maximum Likelihood Estimator

**Table 4:** Confirmatory factor analysis model results by group

Model	*X* ^2^	df	*p*	CFI_cML_	TLI_cML_	SRMR_cML_
**Autistic Teens**						
*Emotional Distress*						
Anger	69.530	5	< 0.001	0.961	0.921	0.074
Anxiety	92.229	20	< 0.001	0.978	0.969	0.050
Depressive Symptoms	61.743	19	< 0.001	0.995	0.993	0.031
Psychological Stress	59.820	19	< 0.001	0.993	0.989	0.034
*Subjective Well-Being*						
Life Satisfaction	85.842	19	< 0.001	0.920	0.883	0.035
Meaning & Purpose	101.802	19	< 0.001	0.984	0.976	0.049
Positive Affect	40.630	18	< 0.001	0.957	0.934	0.030
**General Population Teens**						
*Emotional Distress*						
Anger	35.340	5	< 0.001	0.996	0.991	0.028
Anxiety	73.744	20	< 0.001	0.993	0.991	0.029
Depressive Symptoms	67.167	19	< 0.001	0.997	0.996	0.020
Psychological Stress	88.006	19	< 0.001	0.995	0.992	0.028
*Subjective Well-Being*						
Life Satisfaction	239.379	19	< 0.001	0.962	0.944	0.023
Meaning & Purpose	133.062	19	< 0.001	0.987	0.980	0.040
Positive Affect	170.662	18	< 0.001	0.932	0.894	0.040

*Note* df = Degrees of Freedom, CFI = Comparative Fit Index, TLI = Tucker-Lewis Index, SRMR = Standardized Root Mean Squared Residual, cML = Categorical Maximum Likelihood Estimator

### Measurement invariance

Results of the measurement invariance analyses for the Emotional Distress scales are presented in Table 5. The Depression scale was the only measure that demonstrated metric and scalar invariance, as evidenced by nonsignificant (all *p*’s < 0.01) permutation testing with increasing equality constraints. The Anger and Psychological Stress scales both demonstrated metric invariance but not scalar invariance. Specifically, the chi-square difference test and ∆CFI values for the configural and metric models for the Anger scale were nonsignificant; however, the Anger scale exhibited significant chi-squared (*p =* .009) and ∆CFI values (*p =* .009) when testing for scalar invariance. The Psychological Stress scale also exhibited similar fit between the configural and metric models, but significant deterioration in model fit when testing for scalar invariance, with significant chi squared (*p* < .001) and ∆CFI values (*p* < .001). Finally, the Anxiety scale did not demonstrate metric or scalar invariance based on ∆CFI (*p =* .007).

**Table 5:** Model comparison statistics (w/ permutation) – emotional distress

Model	*Model Comparison Statistics*				
	*Models*	*ΔX* ^2^	*p*	*ΔCFI*	*p*
Anger					
1 – Configural	Null vs. 1	51.956	0.211	0.997	0.156
2 – Metric	1 vs. 2	4.190	0.754	<-0.001	0.854
3 – Scalar	2 vs. 3	14.166	0.009*	<-0.001	0.009*
Anxiety					
1 – Configural	Null vs. 1	62.083	0.841	0.999	0.299
2 – Metric	1 vs. 2	40.601	0.019	− 0.002	0.007*
3 – Scalar	2 vs. 3	-1.831	0.439	0.001	0.615
Depression					
1 – Configural	Null vs. 1	52.711	0.682	1.000	0.461
2 – Metric	1 vs. 2	24.082	0.290	<-0.001	0.222
3 – Scalar	2 vs. 3	-2.044	0.486	< 0.001	0.673
Psychological stress					
1 – Configural	Null vs. 1	56.710	0.042	< 0.999	0.017
2 – Metric	1 vs. 2	14.474	0.488	<-0.001	0.448
3 – Scalar	2 vs. 3	38.374	< 0.001*	<-0.001	< 0.001*

*Note* ΔCFI = Change in Comparative Fit Index, * indicates significant *p*-value < 0.01

Results of the measurement invariance analyses for the Subjective Well-Being scales are presented in Table 6. The Positive Affect scale demonstrated metric and scalar invariance, with nonsignificant chi-square difference test and ∆CFI values between increasingly restrictive models. The Life Satisfaction scale did not demonstrate metric or scalar invariance based on both chi-square difference tests (*p =* .008) and ∆CFI value (*p =* .005). The Meaning & Purpose scale did not demonstrate metric or scalar invariance based significant ∆CFI (*p =* .007).

**Table 6:** Model comparison statistics (w/ permutation) – subjective wellbeing

Model	Model Comparison Statistics				
	Models	ΔX^2^	***p***	DCFI	***p***
Life Satisfaction					
1 – Configural	Null vs. 1	100.734	0.999	> 0.999	0.993
2 – Metric	1 vs. 2	85.488	0.008*	<-0.001	0.005*
3 – Scalar	2 vs. 3	-60.401	0.991	0.001	0.993
Meaning & Purpose					
1 – Configural	Null vs. 1	102.971	0.241	0.998	0.010
2 – Metric	1 vs. 2	52.788	0.020	− 0.001	0.007*
3 – Scalar	2 vs. 3	-21.834	0.922	0.001	0.974
Positive Affect					
1 – Configural	Null vs. 1	103.168	0.295	0.999	0.228
2 – Metric	1 vs. 2	24.401	0.379	<-0.001	0.363
3 – Scalar	2 vs. 3	9.573	0.081	< 0.001	0.086

*Note* ΔCFI = Change in Comparative Fit Index, * indicates significant p-value < 0.01

## Discussion

The current study is the first to document the varying levels of measurement invariance between the self-reported QoL of autistic and general population teenagers across seven PROMIS pediatric scales. Testing measurement invariance is a critical step towards assuring that measures that were developed and validated on non-autistic populations function in the same way for autistic individuals. Our focus on the self-report of teenagers emphasizes the lived experience of QoL across the community-prioritized domains of Emotional Distress (Depression, Anger, Anxiety, and Psychological Stress) and Subjective Well-Being (Life Satisfaction, Positive Affect, and Meaning & Purpose). Such efforts are essential for validating patient-reported outcomes for use in autism research [55]. Taken together, the current study addresses an important gap in the literature on autistic QoL by providing the psychometric validation necessary to confidently use these scales to measure various dimensions of QoL among autistic youth.

Our results highlight varying degrees of measurement invariance on the Emotional Distress and Subjective Well-Being self-report scales for autistic and general population teens. The Depression and Positive Affect scales demonstrated configural, metric, and scalar invariance between the two groups. These results suggest that the constructs are measured in a similar fashion among autistic teens as they are in the general population and that the scores from these scales can be compared meaningfully across autistic and general population groups.

The Anger and Psychological Stress scales both demonstrated configural and metric, but not scalar, invariance between the self-report of autistic teens and general population teens. These results suggest that the scales’ items capture the respective latent constructs across both groups, but researchers should not compare means between autistic and general populations on these self-report scales. The scales demonstrate a degree of measurement bias such that equivalent scores on the scales do not necessarily imply equal levels of anger or psychological stress in autistic versus non-autistic teens. Finally, the Anxiety, Life Satisfaction, and Meaning & Purpose teen self-report scales did not demonstrate metric or scalar invariance between the groups, indicating that these PROMIS scales may function differently between autistic and general population teens. These results suggest that the scales do not capture the intended constructs in autistic teens in the same way as they do in non-autistic teens.

Our findings for varying levels of measurement invariance of the PROMIS scales between autistic and general population teens is not surprising given that these measures were not specifically developed to measure QoL among autistic teens. The PROMIS pediatric Depression, Anxiety, and Anger scales were developed and standardized on a large sample of children and teens recruited from pediatric clinics and school settings [36–38]. Similarly, the PROMIS pediatric Psychological Stress [32], Life Satisfaction [33], Positive Affect [35], and Meaning & Purpose [34] scales were developed and standardized with samples of children from opt-in online panels, school districts, and hospital clinic settings. These samples included children with chronic health conditions, such as asthma, Attention-Deficit/Hyperactivity Disorder, and gastrointestinal disorders; however, autistic children were not reported as a part of the measures’ development samples. The fact that autistic individuals were not knowingly included in these development samples should not negate the tremendous amount of work that goes into creating and validating important patient reported outcome measures to capture different dimensions of QoL in pediatric populations. However, researchers and clinicians using these measures as outcomes in autism research and/or clinical work should do so with the knowledge that most of the PAB-L scales are not specifically validated for use with autistic teens.

To our knowledge, the present investigation is the first study to investigate the factorial validity of the self-report of autistic teens using the PROMIS scales. While previous research has investigated measurement invariance of PROMIS scales between parent-proxy reports for autistic and non-autistic children [25], self-report of QoL domains, particularly among autistic individuals, is likely a better estimation of an individual’s lived experiences of QoL. Future research will benefit from investigations of how PROMIS scales function similarly or differently depending on reporter (e.g., parent-proxy vs. self-report) in capturing autistic QoL. Previous research has highlighted ways in which the measurement of subjective experiences differ between parent-report and autistic adolescent self-report, including social anxiety [56] and sensory sensitivity [57]. Thus, psychometric investigations of QoL measures which include autistic self-report are likely to improve our measurement, and therefore understanding, of autistic QoL.

Beyond the individual scale findings presented, the current investigation highlights an important gap in the field of autism research. Put simply, the rigor with which we measure QoL in autism lags significantly behind the state of measurement science [55]. Specifically, standard psychometric practices, including testing whether a measure that was developed using non-autistic samples functions similarly in autistic populations, are infrequently applied before utilizing the scale in autism research. Such practices are particularly crucial in the adoption of patient-reported outcome measures, including QoL, as these constructs tap into subjective experiences, rather than objective or observable indicators. Researchers using the PROMIS scales have the advantage of a rigorous development and validation process in the general population or for clinical groups that were included in the initial construction. However, this rigorous development process is only advantageous to autism researchers if the scale functions in the same way in autistic populations.

### Clinical implications

The degree that a violation of measurement invariance is problematic depends on how the instrument is used across groups [58]. Some research seeks to compare autistic individuals to non-autistic individuals through comparing scores on a single measure and drawing conclusions based on differences (or lack of differences) between group means. To accurately compare group means, the instrument should demonstrate scalar invariance between the two groups. In the present study, the Depression and Positive Affect scales of the PROMIS self-report demonstrated scalar invariance between autistic and general population teens. Put differently, our results demonstrate that similar scores on the PROMS self-report Depression and Positive Affect subscales imply similar levels of depression and positive affect between autistic and non-autistic teens. As such, these scales can be validly used to compare these groups.

In contrast, other studies may use a teen’s self-report as an outcome within an autistic population, rather than compare across autistic and non-autistic teenagers. In this case, an instrument demonstrating metric (but not scalar) invariance may be sufficient given the research question. Our finding that the PROMIS Anger and Psychological Stress scales demonstrated invariance at the metric level suggests that these scales function similarly in the measurement of anger and psychological stress across groups. However, these scales did not demonstrate scalar invariance, suggesting test bias and that equal observed scores on the PROMIS Anger and Psychological Stress scales do not necessarily imply equal levels of anger or psychological stress in autistic versus non-autistic teens.

### Limitations

Results of our study are limited by several factors related to our samples of autistic and general population teens. First, given that this was secondary data analysis based on virtual surveys, limited information was available regarding the cognitive functioning, verbal abilities, or adaptive behavior skills of our samples. Future work able to characterize more multidimensional characteristics would be helpful in validating the generalizability of results presented here. Relatedly, our current study did not include a representative sample of autistic teens who were reported to be minimally verbal or have co-occurring intellectual disability. It is unknown to what extent our current findings would extend to autistic teens with co-occurring intellectual disability. Given the high prevalence rate of intellectual disability among autistic individuals [59], additional research is necessary to understand how to reliably and validly measure QoL among this population across the lifespan. Finally, autism diagnostic status was not reported in the general population sample data, so it is possible that the general population sample contained some autistic adolescents.

As is common in autism research given the imbalanced sex and gender ratio of individuals diagnosed with autism, our autistic sample had a higher proportion of teens identifying as male than female or transgender. In addition, since only the autistic group was asked about transgender identity, there is no comparison group for transgender adolescents in the general population sample. Future work may benefit from oversampling methods for autistic women, transgender, and nonbinary individuals to characterize their lived experience of QoL. Additionally, both autistic and general population groups were more white and non-Hispanic/Latino than is reflected in the current United States population. Increasing racial and ethnic diversity is largely recognized as a high-priority area of growth across a wide range of autism research [60, 61].

## Conclusion

The results of this study provide evidence that the Depression and Positive Affect scales of the PROMIS can be used confidently in research and clinical work based on the self-report of autistic and non-autistic teens. Scores on the PROMIS Depression and Positive Affect scales can also be compared across these two groups given the psychometric properties of the scales. The PROMIS Anger and Psychological Stress scales demonstrated metric invariance and can be used to measure these QoL constructs in autistic teens, though caution should be used in research seeking to compare autistic and general population teens on these measures. Finally, neither scalar nor metric invariance was indicated for the Anxiety, Life Satisfaction, and Meaning & Purpose scales, suggesting that these scales measure these constructs differently in autistic and non-autistic teens. The inclusion of QoL outcomes is a welcome advancement in autism research; however, the process in validating QoL measures for use in autistic populations is an ongoing process necessary to support such research. We believe the past and current work on the PAB-L exemplifies this process – the PAB-L leveraged rigorously developed PROMIS scales and consulted with autistic individuals, their families, and care providers to select scales that reflect constructs most meaningful for this population. Feasibility, acceptability, and reliability of the PAB-L were favorable [29], and the current work extends this process to examine the psychometric properties of the scales for autistic teenagers as compared with general population teenagers. Multi-group CFAs that demonstrate measurement invariance between autistic and general populations help researchers make empirically supported decisions regarding the measures they use to capture QoL outcomes in their research.

## Electronic supplementary material

Below is the link to the electronic supplementary material.

Supplementary Material 1

## Footnotes

1 ‘Identity-first’ language is preferred by the majority of autistic adults [1, 2] and will be used throughout this manuscript.
