# Supplementary material for: Measurement invariance of the PROMIS emotional distress and subjective well-being domains among autistic and General Population adolescents
Source: Qual Life Res. 2024 Jul 30;33(11):3003–12. doi: 10.1007/s11136-024-03742-9 (PMC11541282; doi:10.1007/s11136-024-03742-9)
Supplement: Supplementary file 1 — Supplementary Material 1 [file 11136_2024_3742_MOESM1_ESM.docx]

| ***Table S1. Collapsed Item Responses*** | | | |
| --- | --- | --- | --- |
| **Scale** | **Item** | **Responses** | **Group Missing** |
| Anxiety | I felt like something awful might happen.  I felt nervous.  I felt scared.  I felt worried.  I worried when I was at home.  I got scared really easy.  I worried about what might happen to me.  I worried when I went to bed at night. | 5 (Almost Always) collapsed with 4 (Often) | General population |
| Anxiety | I worried when I was at home. | 4 (Often) collapsed with 3 (Sometimes) | Autism |
| Anxiety | I got scared really easy. | 4 (Often) collapsed with 3 (Sometimes) | General population |
| Positive Affect | I felt great.  I was in a good mood. | 1 (Never) collapsed with 2 (Rarely) | Autism |

| ***Table S2. Correlated Error Terms in Modified Scales*** | |
| --- | --- |
| **Scale** | **Item Error Terms Correlated** |
| Depression | “I felt alone” and “I felt lonely” |
| Psychological Stress | “I felt stressed” and “I felt that my problems kept piling up” |
| Life Satisfaction | “I was satisfied with my life” and “I was happy with my life” |
| Positive Affect | “I felt calm” and “I felt peaceful” |
|  | “I felt cheerful” and “I felt joyful” |
| Meaning & Purpose | “My life is filled with meaning” and “my life has purpose” |
